# Electron tomography of the nucleoid of *Gemmata obscuriglobus* reveals complex liquid crystalline cholesteric structure

**DOI:** 10.3389/fmicb.2012.00326

**Published:** 2012-09-13

**Authors:** Benjamin Yee, Evgeny Sagulenko, Garry P. Morgan, Richard I. Webb, John A. Fuerst

**Affiliations:** ^1^School of Chemistry and Molecular Biosciences, The University of QueenslandSt. Lucia, QLD, Australia; ^2^Centre for Microscopy and Microanalysis and Institute for Molecular Bioscience, The University of QueenslandBrisbane, QLD, Australia; ^3^Centre for Microscopy and Microanalysis and Australia Institute for Bioengineering and Nanotechnology, The University of QueenslandBrisbane, QLD, Australia

**Keywords:** cholesteric structure, electron tomography, *Gemmata obscuriglobus*, nucleoid, planctomycetes

## Abstract

The nucleoid of the planctomycete *Gemmata obscuriglobus* is unique within the Bacteria in being both highly condensed and enclosed by a double-membrane nuclear envelope, seemingly analogous to the nucleus of eukaryotes. Here we have applied electron tomography to study high-pressure frozen, cryosubstituted cells of *G. obscuriglobus* and found multiple nested orders of DNA organization within the condensed nucleoid structure. Detailed examination of the nucleoid revealed a series of nested arcs characteristic of liquid crystalline cholesteric DNA structure. The finest fibers were arranged in parallel concentrically in a double-twist organization. At the highest order of nucleoid organization, several of these structures come together to form the core of the *G. obscuriglobus* nucleoid. The complex structure of DNA within this nucleoid may have implications for understanding the evolutionary significance of compartmentalized planctomycete cells.

## Introduction

Planctomycetes are bacteria with many unusual features including compartments (Lindsay et al., 2001; Fuerst and Sagulenko, 2011), and a condensed nucleoid which differs from nucleoids of other bacteria, typically found in a diffused coralline state without any apparent higher order, when cryofixation processing for electron microscopy (EM) is applied (Bohrmann et al., 1991). The nucleoid may be condensed during specific phases of life cycle such as stationary phase or during sporulation (Setlow et al., 1991; Frenkiel-Krispin et al., 2004). At present, no planctomycetes are known to produce spores so there is not likely to be any correlation of the condensed structure to sporulation. The study of the life cycle of *Gemmata obscuriglobus* by Lee et al. (2009) was comprehensive enough to suggest that the condensed structure could more than likely have persisted throughout the active life cycle. The persistence of a condensed nucleoid throughout a life cycle has implications with respect to genome organization, perhaps at a deeper level than is presently known to exist within the seemingly disordered structure of the *E. coli* nucleoid (Thanbichler et al., 2005). A recent study of *G. obscuriglobus* has not only reiterated the distinctiveness of the intracellular organization and condensed nucleoid in planctomycetes, but also suggests a role of the compacted chromatin in ability of these bacteria to survive lethal doses of ionizing radiation (Lieber et al., 2009).

The recent use of electron tomography has been an invaluable tool in the study of macromolecular structures within cells, including the study of anammox planctomycetes (van Niftrik et al., 2009). In order to understand how cellular processes such as transcription and translation occur in the planctomycetes where the nucleoid is compartmentalized within internal membranes, we need a better understanding of DNA organization. In this study, we used electron tomography techniques combined with high-pressure freezing to examine the fine structure of the condensed nucleoid structure of *G. obscuriglobus* and revealed a complex organization of the nucleoid consisting of several levels of DNA compartmentalization.

## Materials and methods

### High-pressure freezing and cryosubstitution

Five to seven-days old cells of *G. obscuriglobus* were high-pressure frozen and cryosubstituted according to methods described in Lee et al. (2009) and Lonhienne et al. (2010), in a solution of 2% osmium tetroxides and embedded in Lowicryl HM20 resin. Samples were then cut into 300 nm serial sections using a Leica EM UC6 ultramicrotome (Leica Microsystems, Austria) and stained with 2% uranyl acetate in water and lead citrate for the 300 nm tomography sections. Ten nanometer protein-A gold particles were used as fiducial markers on both surfaces whenever possible to aid in subsequent reconstruction of the tomograms.

### Dual-axis tilt-series

Tilt-series data of whole cells were collected at 23,000× magnification using a Direct Electron LC1100 4k × 4k camera (Direct Electron, USA) on an FEI Tecnai F30 (FEG) TEM (FEI Company, The Netherlands) over a tilt range of ± 66° at 1.5° increments for the A-axis and 3° increment for the B-axis, using SerialEM software (The Boulder Lab for 3D Electron Microscopy of Cells, USA).

### Tilt-series reconstruction

Tilt-series micrographs of whole cells of *G. obscuriglobus* were reconstructed with the R-weighted back projection algorithm using IMOD/Etomo software (The Boulder Lab for 3D Electron Microscopy of Cells, USA) and segmented using IMOD's automated isosurface rendering function.

Dual-axis tilt-series of 300 nm thick serial sections of cryosubstituted, resin-embedded *G. obscuriglobus* were acquired and seven tomograms were generated, resulting in the reconstruction of an entire single cell.

## Results

**Figure 1:**
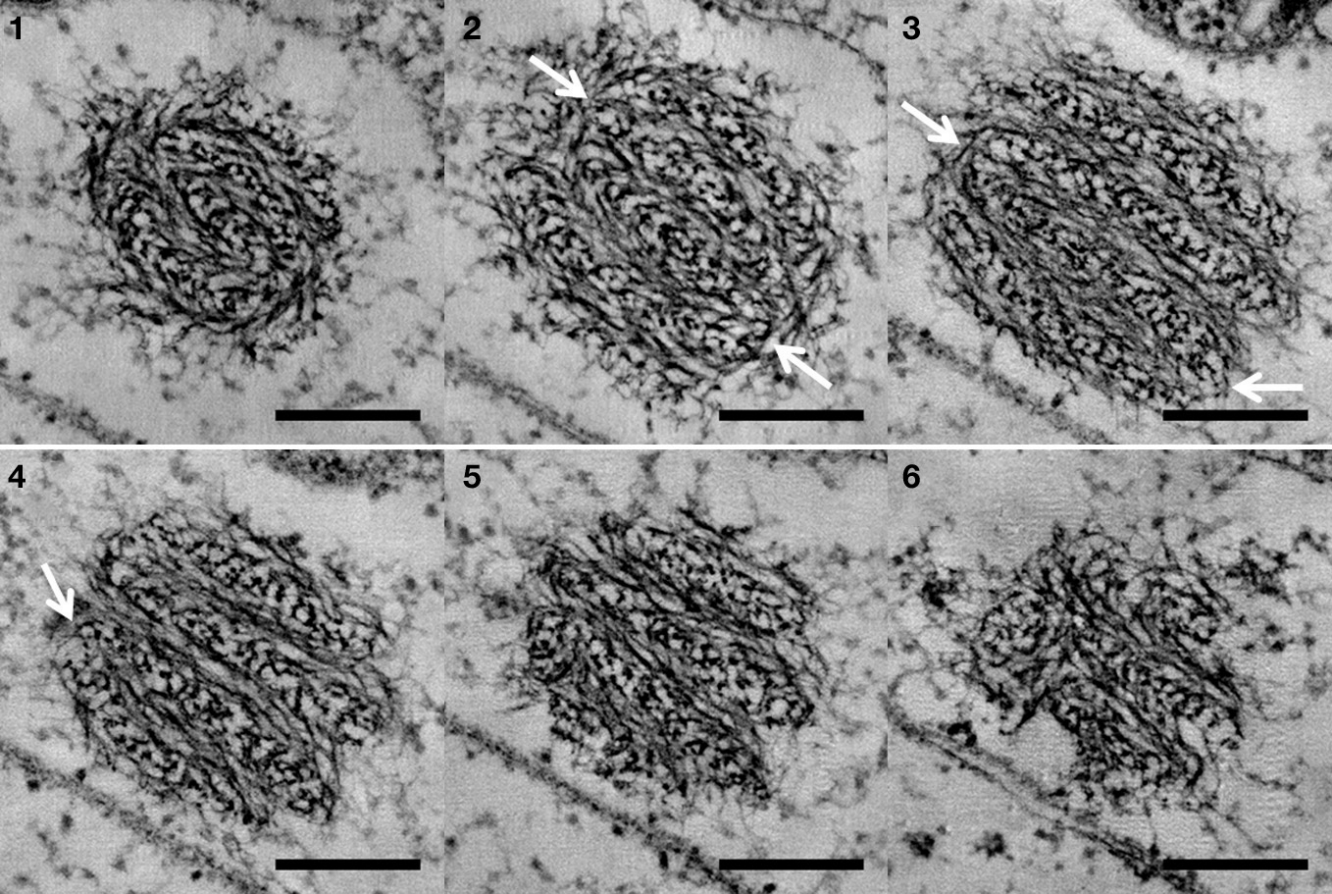
**Sequential display of cross-sections of the whole nucleoid structure derived from tomography of a tilt-series of thick sectioned cryosubstituted *G. obscuriglobus*.** Frames **1–6** represent sequential frames of a tilt-series through the same nucleoid. A banding pattern formed by electron dense layers of compacted chromatin and translucent layers of thin nucleofilaments was observed to persist through the serial sections. White arrows show regions of connections between adjacent layers, suggesting that the layers form one continuous structure. Scale bar, 200 nm.

**Figure 2:**
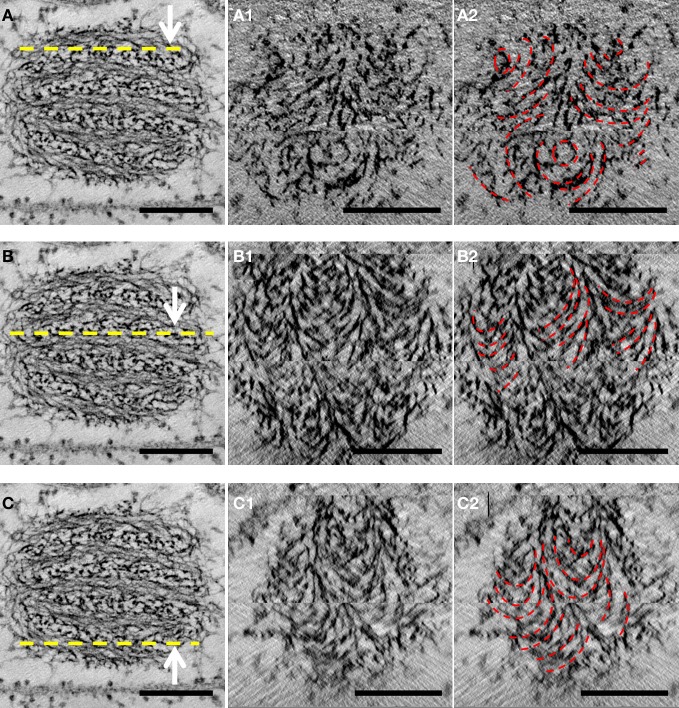
**Longitudinal cross-section views of the nucleoid structure derived from tomography of tilt-series of thick sectioned cryosubstituted *G. obscuriglobus*.** (Left column: **A,B,C**) The yellow dotted line marks the region at which a cross-sectional view was obtained for detailed analysis of chromatin organization. Each numbered frame represents a view of 10 tomographic slices corresponding to the level of the nucleoid structure shown in the corresponding left column by the dotted line. Each view represents a 12.4 nm section through the nucleoid. Concentric rings can be seen in **A1** and outlined in red in **A2,** and series of parallel arcs can be seen in **B1** and **C1** and outlined in red in **B2** and **C2**. These patterns are outlined in red in panels in the right column. Scale bar, 200 nm.

**Figure 3:**
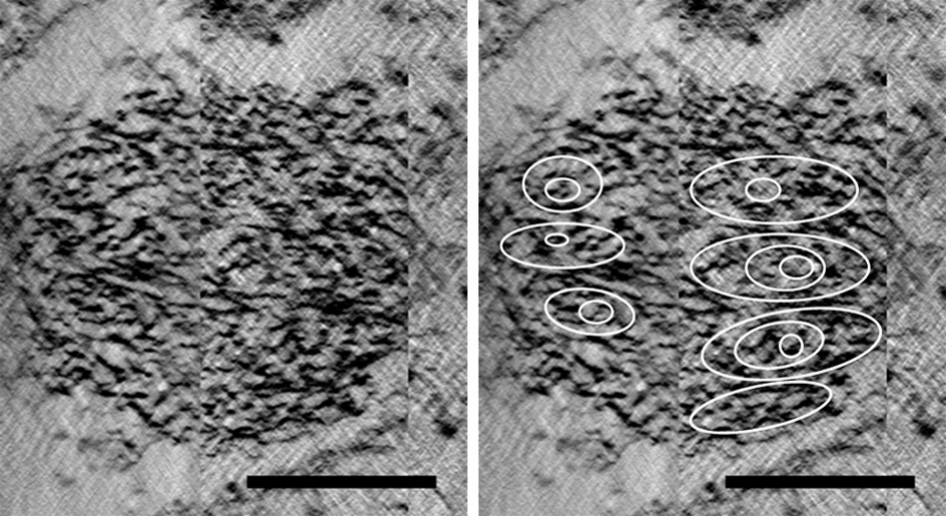
**(Left)** Latitudinal cross-section view of the nucleoid structure derived from tomography of tilt-series of thick sectioned cryosubstituted *G. obscuriglobus*. **(Right)** Same cross-section view but with the stacks of concentric circles corresponding to dense layers present within the nucleoid marked out in white Scale bar, 200 nm.

Reconstruction of whole cells of *G. obscuriglobus*, in particular the nucleoid, showed the nucleoid to be condensed into a roughly spherical structure. In the cell shown in Figure 1, this nucleoid is approximately 570 nm in diameter. Serial sections of the nucleoid (Figure 1) clearly show the periodic organization of electron-dense layers of higher order structure interspaced by thin nucleofilaments of lower electron density in nature. Longitudinal cross-section view of the top section of the nucleoid, as shown in Figure 2A, revealed a number of spiral patterns, forming concentric ring structures up to approximately 200 nm in diameter (Figure 2A1), and outlined in red for better visualization (Figure 2A2). A planar view through the middle section of the nucleoid showed three separate sets of parallel arcs along rod-shaped structures with a diameter between 120 and 180 nm (Figures 2B1,B2) with a periodicity of approximately 20 nm between each arc and the next arc. The outlines of these arcs are more distinct in the cross-sectional view of the bottom section of the nucleoid (Figures 2C1,C2). A latitudinal cross-section view showed the concentric pattern arranged in stacks one on top of another (Figure 3). Measurements of the thickness of one of these stacks correspond to the thickness of the electron dense layers seen in Figure 1.

**Figure 4:**
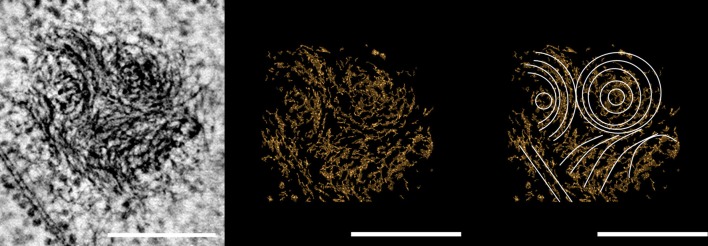
**Micrograph derived from tomography of tilt-series of a thick section of a dividing cell of cryosubstituted *G. obscuriglobus.*** The solenoid arrangement of the spiral structures in the nucleoid can be seen in the micrograph **(Left)**. Note the series of concentric circles and “nested arches” (white) characteristic of liquid crystalline DNA organization, highlighted in the computer graphics versions of the image (**Middle** and **Right**). Scale bar, 200 nm.

**Figure 5:**
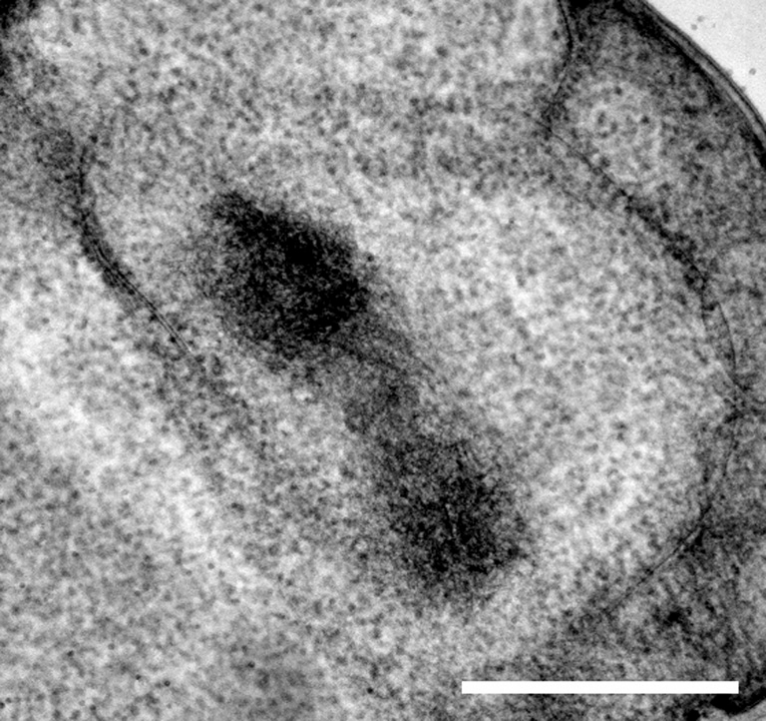
**Composite image of 500 tilt-series image slices through a thick-sectioned cryosubstituted *G. obscuriglobus* cell.** The difference in density between the highly dense “core” at the two ends and the less dense “bridge” between them seems to suggest that the chromosome has been captured during replication. Scale bar, 500 nm.

We also obtained images for the nucleoid of *G. obscuriglobus* during replication. Similar spiral patterns can clearly be seen from the tomographic slice of one of the nucleoids and we can clearly see at least three such spiral structures, one of which appears to be the series of concentric rings, while another shows a series of parallel arcs (Figure 4). As one progresses through the sections, one of these spirals appears to be either winding up from, or unwinding toward a second similar nucleoid structure, though it is not possible to determine the actual direction of movement between the two nucleoid structures. The bundles of DNA filaments in transition appeared in lower contrast, in stark comparison to those within the dense core of the two nucleoids at either end of the structure (Figure 5).

**Figure 6:**
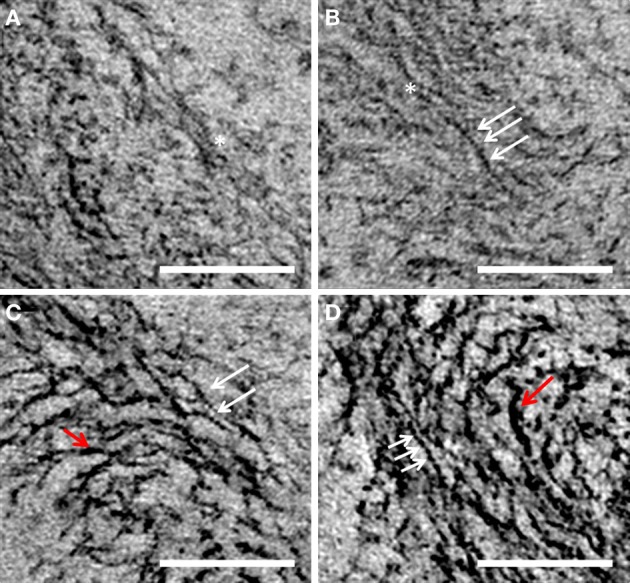
**A, B, C, and D are different views of nucleoid fibrils of *G. obscuriglobus* taken from tilt-series micrographs of thick-sectioned cryosubstituted cells.** White arrows show the presence of putative protein complexes along the length of DNA filaments. Red arrows show the thicker filaments. Asterisk marks out the location where one filament is connected laterally to another adjacent filament forming the next “thick filament” level of nucleoid organization. Scale bar, 100 nm.

In certain regions of the nucleoid there was sufficient resolution to distinguish finest resolvable filaments from bundled filaments. Electron-dense bead-like particles appeared to be attached to the finest filaments (Figure 6B), several of which could be seen aligned to each other (Figure 6A), forming the basis for the next level of thick filament organization (Figures 6C,D). Certain filament bundles in the transitional area appeared to be either in the process of being bundled together or in the process of being separated, as indicated by the white asterisk in Figures 6A and B.

## Discussion

Under high-resolution EM of cells sectioned after cryosubstitution processing for EM, the nucleoid of bacteria such as *E. coli* is often observed to have a coralline distribution, occupying a ribosome-free area within the cell space (Bohrmann et al., 1991). When the cell organization of *G. obscuriglobus* was first investigated using ultrastructural methods, the nucleoid was observed to be highly condensed and enclosed by a double-membrane nuclear envelope (Fuerst and Webb, 1991), features seemingly analogous to the nucleus of eukaryotes. Advances in cryopreparation of samples for electron microscopy such as cryosubstitution involving cryofixation rather than chemical fixation as first fixation stage have progressed sufficiently over the years so as to achieve elimination of artificial aggregation of the nucleoid due to chemical fixation (Bohrmann and Kellenberger, 2001), and these advanced techniques confirm the condensed nature of *G. obscuriglobus* nucleoid.

Electron tomography performed on whole cells of *G. obscuriglobus* via tilt-series from thick sections viewed in a high-voltage electron microscope made it possible to model the entire nucleoid structure in three-dimensions. The nucleoid could be examined in fine detail so that higher order structure was discerned, especially with respect to the condensation of the nucleoid itself.

### Fine structure of the *G. obscuriglobus* nucleoid

One of the main differences between the chromatin of eukaryotes and bacterial chromosome lies at the level of the DNA filaments, whereby histone complexes organize DNA filaments into nucleosomal structures, whereas bacterial chromosomes are devoid of such nucleosomal complexes. In our study, we observed that DNA filaments within the *G. obscuriglobus* nucleoid were interspersed with electron-dense bead-like particles along its length, which could be some kind of DNA-binding protein or complex of proteins, potentially representing some form of DNA organization at the filament level. Bioinformatics study of the draft genome of *G. obscuriglobus* had failed to find any discernible homolog of histones, although two unique sequences of the bacterial HU-proteins were uncovered (Yee et al., 2011). The *in silico* discovery of these DNA-binding proteins is an indication that some kind of nucleoid-associated proteins might exist in *G. obscuriglobus* and could possibly be correlated to the observation of the electron-dense particles along the DNA filaments. However, identification of these electron-dense particles would require future study involving isolation and identifying them via proteomics methods. Their potential relation to HU proteins found from *in silico* analyses could be determined by use of immunogold electron microscopy based on antibodies raised using expressed and purified HU proteins as antigens. The search for sequences homologous to histones in the genome of *G. obscuriglobus* is hindered by the lack of full genome data for *G. obscuriglobus* at the moment. However, genomic analysis of other planctomycetes has not so far detected any sequence homologs of eukaryote histones (Glockner et al., 2003; Labutti et al., 2010; Goker et al., 2011). Planctomycetes may achieve chromosome compaction via a different approach other than the use of recognizable eukaryotic nucleosome structures. Of course planctomycetes divergence relative to other organisms is such that eukaryote-like histone fold proteins or other condensing proteins may yet be annotated from genome sequence data. BLAST searches alone are known to have limitations for this group (Fuerst and Sagulenko, 2011).

An association of DNA with liquid crystals was found during the study of the chromosomes of dinoflagellates, which describes the spontaneous formation of ordered phases from a high concentration of a semi-rigid polymer such as DNA (Bouligand et al., 1968; Gautier et al., 1986). The series of nested arches formed by the DNA filaments observed in *G. obscuriglobus* are structurally similar to the DNA filaments observed in dinoflagellates, which in turn resemble the distinctive ordered arrangements of molecules rotated at a constant angle on a pseudoplane characteristic of a cholesteric liquid phase (Bouligand et al., 1968; Rill et al., 1989). The term “cholesteric” stemmed from the observation of liquid crystal phases formed by solutions of cholesterol derivatives, and the periodicity and parallel nature of the filaments observed in the nucleoid of *G. obscuriglobus* are characteristic of the ordered phase of liquid crystalline organization as well (Ginsburg et al., 1984). The condensed and liquid crystalline organization of the nucleoid of *G. obscuriglobus* is consistent with that found in a previous study of cell structure and radiation survivability of *G. obscuriglobus* (Lieber et al., 2009). The cholesteric form is just one of many conformations of DNA condensation, most of which are associated with eukaryotic chromatin, as reviewed by Livolant (1991). Similar nucleoid structure has also been observed in other bacteria, usually as a conformational change during stationary phase; examples include the nucleoid of the radio-tolerant bacterium *Deinococcus radiodurans* and nucleoid of *E. coli* cells treated with chloramphenicol (Eltsov and Dubochet, 2005).

The dinoflagellates are a group of primitive unicellular algae, the nuclei of which remain condensed throughout most of their life-cycle. Despite being a eukaryote, the nuclear filaments in dinoflagellates are devoid of nucleosome-like particles (Gautier et al., 1979, 1986; Rizzo and Burghardt, 1980). Although core histones have been detected in low levels, it has been suggested that these proteins have alternative roles other than being involved in nucleosome assembly (Hackett et al., 2005; Roy and Morse, 2012). Similarly, proteins similar to those of prokaryotic histone-like proteins have also been found in low concentrations within dinoflagellate chromosomes (Rizzo and Nooden, 1972; Wong et al., 2003). However, the low protein to DNA molar ratio makes it unlikely for proteins to be solely responsible for the condensation of the dinoflagellate chromosome; instead the condensation might be due to a combination of entropy-driven force derived from the twisted nature of the dinoflagellate chromosome (Chow et al., 2010), followed by the neutralization of negative charge of DNA by abundance of divalent cations to achieve maximal DNA compaction (Levi-Setti et al., 2008). In the early model proposed by Bouligand et al. (1968), the widely accepted representation for the structure of dinoflagellate chromosomes, stacks of discs are proposed with DNA filaments on each disc running parallel to each other. However, the nucleoid of *G. obscuriglobus* seems to differ from this arrangement in the alignment of filaments, since in a transverse section view of the nucleoid it was observed to have an organization more akin to concentric circles instead. Such concentric rings were previously described by Eltsov and Zuber (2006) to explain the double-twist DNA arrangement of localized bacterial DNA bundles, in which a solenoid configuration is formed from the helical arrangement of DNA molecules wrapping around a central core. There are also such comparable arrangements in eukaryotic cells such as those of the human retina, where parallel 30 nm chromatin fibers have been visualized coiling around a nuclear axis in electron micrographs (Diaz, 1983). Considering that multiples of such concentric rings were observed to be adjacent to each other, essentially forming the core of the *G. obscuriglobus* nucleoid, the overall structure bears more resemblance to the higher order of eukaryotic chromatin arrangement than the local DNA bundles of bacteria.

### Structural organization of the *G. obscuriglobus* nucleoid

**Figure 7:**
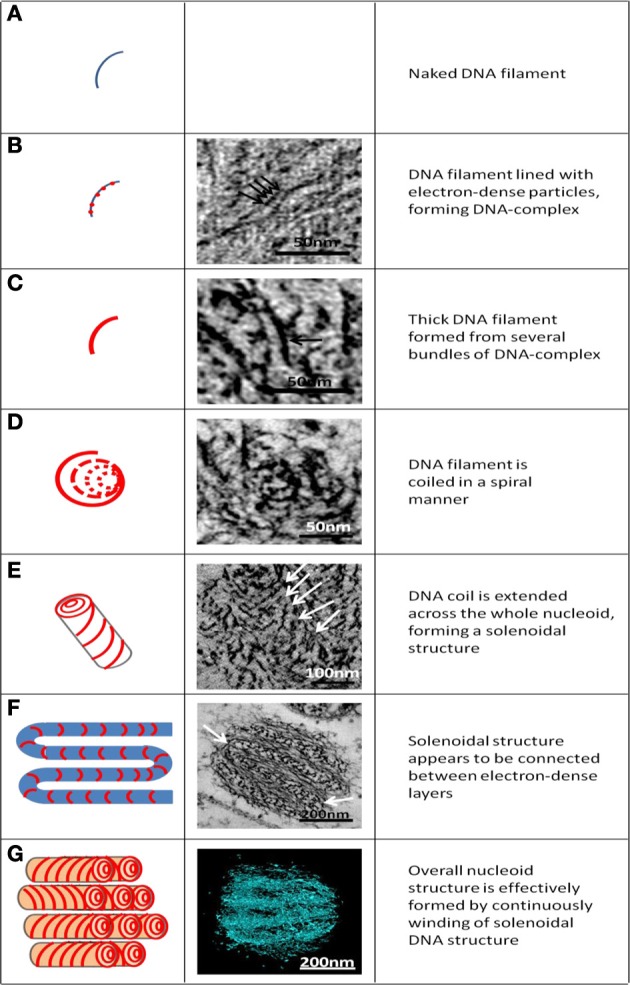
**Hypothesized levels of structural organization of the *G. obscuriglobus* nucleoid, seen in schematic diagram (left column) and corresponding micrographs (B–F) and reconstruction from tomography of the nucleoid (G). (A)** The naked DNA helix assumed as the first level of structure. **(B)** DNA helix with associated bound protein beads (the “DNA-protein complex”). Arrows indicate individual beads. **(C)** Thick filament (arrow) formed from parallel arrangement of individual beaded DNA-protein complex filaments. **(D)** Coil of thick filaments arranged in a spiral or helix. **(E)** Coil extended in three dimensions along the nucleoid forms a solenoid (coiled coil) structure indicated by downwards spiral pattern in the micrograph (arrow). **(F)** Solenoidal structures are connected between electron dense layers (arrow). **(G)** Overall arrangement of DNA within the nucleoid due to continuous and connected winding of the solenoidal structure seen in schematic diagram e. In three dimensions, the stacks seen in the schematic diagram will connect to correspond to there construction.

On-going genome sequencing of *G. obscuriglobus* (NCBI NZ_ABGO00000000.1) has provisionally annotated the size of the genome at 9.16 Mbp (mega base pairs), a figure approximately double than the genome of *E. coli* taken as 5 Mbp. Based on measurements made in the current study, we estimate the volume of the roughly spherical *G. obscuriglobus* nucleoid structure to be ca. 0.1 μm^3^, approximately half the volume of 0.2 μm^3^ reported for the volume of the *E. coli* nucleoid (Skoko et al., 2006). This results in a compaction ratio (genome length/nucleoid volume) of the *G. obscuriglobus* nucleoid of at least four times that of the *E. coli* nucleoid. This signifies a high degree of compaction of the *G. obscuriglobus* nucleoid. It is therefore not surprising that some form of ordered arrangement should exist to organize the huge concentration of DNA into a condensed structure. Therefore, we propose the following sequential structural organization of the *G. obscuriglobus* nucleoid likened to a ball of wool formed by intricate bundling of DNA filaments. As illustrated in Figure 7, at the DNA filament level, naked DNA filaments **(A)** are lined with protein complexes in an interspersed manner **(B)**, and several of such filament-complexes are bundled together to form the thick filaments at the next level of organization **(C)**. These thick filaments are then coiled round a hypothetical center to give the appearance of solenoidal structure resembling a long hollow rod **(D** and **E)**. This long hollow rod, representing the whole genome of *G. obscuriglobus*, is then bent or twisted at regular intervals to give the appearance of a layered structure **(F)**. The continuous and connected winding of the solenoidal structure into stacks will give rise to a three-dimensional nucleoid structure showed in **(G)**.

Since the *Gemmata* nucleoid is highly condensed, the underlying implication is that there must also be some form of counteracting force to prevent aggregation of DNA at high density, such as the nucleosome organization observed in eukaryotes (Minsky et al., 1997), or proteins similar to the architectural proteins in Bacteria and Archaea (Browning et al., 2010). Alternatively, or even in concurrent with protein-mediated condensation, the presence of metal cations such as Mg^2+^ and Ca^2+^ stabilizing the nucleoid structure might be sufficient to compact the nucleoid, as in the case of dinoflagellates (Levi-Setti et al., 2008). It would be useful to examine the cation distribution in the nucleoid of *G. obscuriglobus* to determine the extent in which cations assist in the condensation of the nucleoid.

### Implications for a condensed nucleoid

In its coralline state, the *E. coli* chromosome is structurally constrained via negative supercoiling (Stuger et al., 2002). Nucleoid-associated proteins are known to target intergenic regions, may be involved in gene regulation and may also determine topological domain barriers (Sinden and Pettijohn, 1981; Grainger et al., 2006; Noom et al., 2007; Dorman, 2010). Being highly condensed in a liquid crystalline state, the nucleoid of *G. obscuriglobus* might be expected to be effectively a transcription-inert structure, and perhaps highly limiting for the access of RNA-polymerases to genes within the condensed core. However, although there is currently no information on the localization of RNA-polymerases nor single-stranded DNA within cells of *G. obscuriglobus*, the detection of RNA on the periphery of the nucleoid (Lindsay et al., 2001) coincides with what is known for *E. coli*—that metabolically active DNA is found at the nucleoid boundary (Hobot et al., 1987). These two seemingly conflicting views provisionally establish the organization of the nucleoid of *G. obscuriglobus* into two different states; with part of the structure such as the electron-dense layers possibly being metabolically inert whereas DNA in the periphery is involved in active transcription. Superficially, these two states of *G. obscuriglobus* nuclear DNA are not unlike the highly condensed heterochromatin and the transcriptionally active euchromatin of the eukaryote nuclei although analysis of the gene content of the two respective states will be required for further comparison. More importantly, dynamic chromatin structures such as euchromatin and heterochromatin have been shown to be involved in the repair of double-strand breaks in DNA (Downs and Cote, 2005; Xu and Price, 2011) and gene organization within these two states could thus potentially provide insight to the radioresistance of *G. obscuriglobus*. There might be a lack of direct correlation between liquid crystalline order of DNA and radioresistance, but the possibility remains open that underlying factors such as genome organization coupled with chromatin architecture could be an essential basis for radiation survivability along with suitable DNA-repair systems.

Although the mechanisms by which *G. obscuriglobus* compacts its nucleoid are not yet known, it is perhaps just as important to consider the evolutionary forces that may have driven the formation of such a divergent nucleoid structure. An increase in localized DNA density due a large genome size (9 Mb) and the compartmentalization of the nucleoid by a membranous envelope may be the factor at play in *G. obscuriglobus*. Other planctomycete species with smaller genomes and simpler compartmentalized cell plans, e.g., *Pirellula staleyi* and *Blastopirellula marina* (Lindsay et al., 2001), are likely subjected to a similar increase in DNA density within the riboplasm due to formation and enclosure by the intracytoplasmic membrane, which may also explain the condensed nucleoids in cells of those species. An analogous mechanism could explain condensed chromosomes of dinoflagellates as well, where endosymbiotic gene transfer and lateral gene transfer from plastids and symbionts has been proposed to be result in an increased genome size of dinoflagellates (Wisecaver and Hackett, 2011), and a correlated extremely high density of DNA in dinoflagellates and associated chromosome restructuring (Kellenberger and Arnold-Schulz-Gahmen, 1992). The increase in DNA density could also have affected the functionality of commonly used architectural proteins such as histones, which do not seem to participate extensively in the structuring of the chromosome (Hackett et al., 2005; Roy and Morse, 2012). As such, alternative methods such as the use of metal cations (Herzog and Soyer, 1983; Levi-Setti et al., 2008), or the use of the histone-like proteins obtained through lateral gene transfer (Wong et al., 2003) may have resulted in a different form of nucleoid compaction. The divergent HU proteins identified in *G. obscuriglobus* (Yee et al., 2011) are perhaps an indication that this species uses proteins or condensation processes which are different from those employed by other bacteria.

Lastly, chromosome segregation in the compartmentalized cells of *G. obscuriglobus* may involve special mechanisms in which condensed nucleoids within a closed nuclear body may participate in segregation and cell division directly, since there may be no need for connection with cytoplasmic membrane during segregation (suggested by the appearance of the cell in Figure 5).

The evolutionary scenarios suggested are purely speculative but serve to provide a working hypothesis that the nucleoid of *G. obscuriglobus* is a unique structure formed as a result of unconventional proteins as well as mechanisms utilized by other bacteria. Future experiments would include testing the possible roles of metal cations in DNA compaction independent of condensing proteins as has been suggested to occur in dinoflagellates (Levi-Setti et al., 2008), and examining the nucleoid of other planctomycetes as a form of comparison and confirmation of shared features of the unique structure of the planctomycetes nucleoid. In addition, the use of specific antibodies to *G. obscuriglobus* HU proteins combined with the tomographic reconstruction of the nucleoid could determine the distribution of these proteins and provide insight into their association with the nucleoid.

## Conclusion

The limitation of using a single two-dimensional micrograph, often used to display internal structure of cells of *G. obscuriglobus*, is the inability to fully demonstrate the structure of the condensed nucleoid. By performing electron tomography using high-voltage TEM on serial thick sections of a single cell of *G. obscuriglobus*, and reconstructing the resulting tomograms of the whole cell, we have revealed the organization of DNA filaments within the condensed nucleoid. While examining the fine structure of the nucleoid, we observed complexes along uncondensed DNA filaments that we interpret as possible protein complexes, which while they do not resemble histone complexes, nevertheless suggest that nucleoid-associated proteins might have a role in the condensation of *G. obscuriglobus* nucleoid. We have proposed a step-wise model by which DNA is organized via the action of coiling and bending to gradually form higher order arrangements, and ultimately compact the nucleoid.

### Conflict of interest statement

The authors declare that the research was conducted in the absence of any commercial or financial relationships that could be construed as a potential conflict of interest.
